# Gamma Irradiation of Poly(lactide‐co‐glycolide) Scaffolds Reduces the Mechanical Stability and Function of Islet Grafts in Diabetic Nonhuman Primates

**DOI:** 10.1002/bit.70134

**Published:** 2025-12-24

**Authors:** Jessica L. King, Christopher Spencer, Richard Youngblood, Kelly Crumley, Elizabeth Bealer, Peter D. Rios, Ira Joshi, Sofia Ghani, Douglas Isa, James J. McGarrigle, David Cook, Conor Locke, Adam Abraham, Andrea Clark, José Oberholzer, Lonnie D. Shea

**Affiliations:** ^1^ Department of Biomedical Engineering University of Michigan Ann Arbor Michigan USA; ^2^ CellTrans, Inc. Chicago Illinois USA; ^3^ Department of Orthopaedic Surgery University of Michigan Ann Arbor Michigan USA; ^4^ Department of Visceral and Transplant Surgery University of Zurich Zürich Switzerland; ^5^ Department of Surgery University of Michigan Ann Arbor Michigan USA

**Keywords:** islet transplantation, microporous scaffolds, type 1 diabetes

## Abstract

Clinical islet transplantation has long been investigated as a potential cure for type 1 diabetes (T1D), yet standard intrahepatic delivery leaves islets prone to an instant blood‐mediated inflammatory response. Herein, we investigated the design of microporous poly(d,l‐lactide‐co‐glycolide) (PLG) scaffolds for extrahepatic islet transplantation in mouse and nonhuman primate (NHP) models. Acellular scaffolds elicited only a mild inflammatory response following implantation into the omentum. On scaffold islet transplantation had extensive insulin staining at 4 weeks yet modest insulin requirement reductions in diabetic NHP recipients. Scaffolds were sterilized by irradiation and exhibited fragility during seeding and implantation, motivating an increase in the manufacturing ratio of PLG:NaCl from 1:30 to 1.25:30 w/w. These scaffolds exhibited no differences in porosity or interior geometry between sterilization conditions, and transplants in mice restored normoglycemia. We piloted a modified scaffold study in a fourth NHP, and although scaffold integrity was improved, the transplant outcome was similar. We subsequently tested intermediate PLG:NaCl ratios in mice, finding that a 1.15:30 ratio achieved a balance of mechanical stability and islet compatibility. Overall, these studies identify that scaffold porosity can be adjusted to account for the impact of sterilization on transplantation.

## 1 Introduction

Type 1 diabetes (T1D) is an autoimmune disease characterized by the destruction of the insulin‐producing pancreatic β cells, affecting ~8.4 million people worldwide (Gregory et al. 2022; Zajec et al. 2022). Patients administer exogenous insulin through manual daily injections or an insulin pump, and even with diligent management, patients are at heightened risk of chronic cardiovascular and microvascular complications, in addition to acute conditions such as ketoacidosis and hypoglycemia (Joish et al. 2020; American Diabetes Association 2023). The global T1D patient population is expected to increase to 13.5–17.4 million by 2040, (Gregory et al. 2022) motivating the development of improved therapies such as islet transplantation.

Islet transplantation offers a promising alternative to current treatments due to its potential to functionally restore euglycemia with little or no exogenous insulin requirement. In 2023, Lantidra (donislecel) became the first FDA‐approved allogeneic pancreatic islet cell therapy (Parums 2023; Qi et al. 2014). In Phase 1/2 clinical trials, 60% of Lantidra recipients maintained insulin independence 5 years posttransplant (Qi et al. 2014). However, the therapy has multiple challenges, including proneness to the instant blood‐mediated inflammatory reaction in the liver, risks and side effects associated with immunosuppression, and the irretrievability of the graft (Qi et al. 2014; Czarnecka et al. 2023; Naziruddin et al. 2014; Williams et al. 2018). Vertex Pharmaceuticals and Sernova Corp have ongoing clinical trials employing intrahepatic infusion and macroencapsulation of cells, respectively (ClinicalTrials.gov 2023a, 2023b). Despite this progress, only a subset of the population living with T1D has a suitable risk–benefit profile to receive these treatments, underscoring the need for improving transplantation safety and efficacy (Parums 2023; Qi et al. 2014; ClinicalTrials.gov 2023a, 2023b).

Several extrahepatic sites have been investigated for islet transplantation, such as the subcutaneous layer, bone marrow, skeletal muscle, gastric submucosa, anterior chamber of the eye, and intraperitoneal fat (Brusko et al. 2021; Arifin et al. 2016). Extrahepatic sites offer the opportunity to locally control the transplant microenvironment to promote engraftment and long‐term function while minimizing or eliminating systemic nonspecific immunosuppression with local immunomodulation or operational tolerance induction (Gibly et al. 2011). The omentum is of particular interest due to its superficial location relative to internal organs, as well as its rich vasculature and expansive volume. Several groups have transplanted allogeneic islets in the omentum (Berman et al. 2009; 2016; Baidal et al. 2017; Stice et al. 2018; Lei et al. 2022; Deng et al. 2023). Most recently, euglycemia and insulin independence were achieved in a 1:1 donor to recipient model by distributing islets suspended in a plasma‐thrombin gel across the omentum of cynomolgus monkeys (Deng et al. 2023). These findings demonstrate the suitability of the omentum as a transplant site, and biomaterial platforms for cell delivery can support their engraftment at extrahepatic sites (Brusko et al. 2021; Berman et al. 2009, 2016; Baidal et al. 2017; Stice et al. 2018; Lei et al. 2022; Deng et al. 2023; Hunckler and García 2020).

In this study, we investigate the design of microporous, poly(d,l‐lactide‐co‐glycolide) (PLG) scaffolds for intra‐omental islet transplantation, with studies performed in nonhuman primates (NHPs) and mice as a step toward translation (Gibly et al. 2011; Blomeier et al. 2006; Gibly et al. 2013; Kasputis et al. 2018; Youngblood et al. 2019). Scaffolds were fabricated with a 35 mm diameter to account for the large islet demand in NHPs. Acellular scaffolds were initially transplanted into the omentum of healthy NHPs to assess the inflammatory response. Subsequent studies were performed with allogeneic islet transplants into diabetic NHPs, with monitoring of blood glucose, C‐peptide, insulin administration, and cell survival. These studies identified a decreased mechanical stability for scaffolds that had been sterilized by γ irradiation. The PLG to salt porogen ratio was modified, with characterization of the scaffold and additional mouse and NHP transplants. These studies demonstrated the tunability of scaffold manufacturing to compensate for the effects of γ irradiation, with future studies needed to demonstrate efficacy in a large animal model.

## 2 Results

### 2.1 Human Islets and NHP Islets on Scaffolds Effectively Lower Blood Glucose in Diabetic Mice

**Figure 1:**
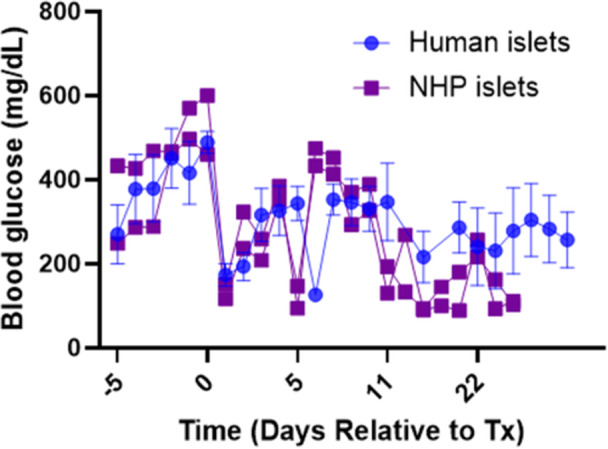
Comparison of glycemic control between mice transplanted with 2000 IEQ human islets (*n* = 4, mean and SEM shown) and nonhuman primate islets (*n* = 2) on nonirradiated scaffolds fabricated with a 1:30 w/w PLG:NaCl ratio.

We confirmed our established scaffold‐islet transplantation approach using NHP‐derived islets, with human islets as a control, in order to support subsequent studies in NHPs. Four streptozotocin (STZ)‐induced diabetic NSG mice received a 2000 IEQ (±20%) total dose of human islets on two scaffolds in the epididymal fat pads, and two mice received a 2000 IEQ (±20%) total dose of NHP islets using the same approach. After transplantation, all transplant recipients had reduced fasting blood glucose levels, with recipients of NHP islets achieving sustained euglycemia (< 200 mg/dL) beginning at Day 10 posttransplantation (Figure 1).

### 2.2 Acellular, Irradiated Scaffolds Elicit Minimal Immune Response in NHPs After 30 Days

**Figure 2:**
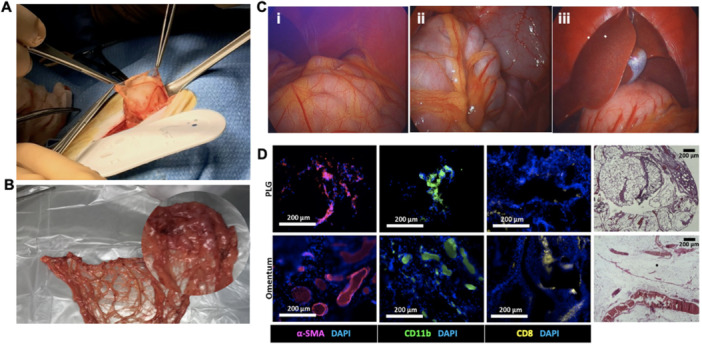
Immunoreactivity study of acellular, irradiated PLG scaffolds, fabricated with a 1:30 PLG:NaCl ratio, in cynomolgus monkeys. (A) Visualization of scaffold insertion in the omentum. (B) Entire excised omentum, with the location of the PLG scaffold magnified. (C) Necropsy images. (i) Gross view of the omentum with no fibrotic tissue visible. (ii) Gross view of the omentum overlaying the colon. (iii) Gross view of the liver, gallbladder, and surrounding viscera. (D) Representative staining of sections from the PLG scaffold (bottom row) and omentum (top row) for α‐SMA (fibroblasts), CD11b (macrophages), and CD8 (cytotoxic T cells). Hematoxylin and eosin staining was also performed. All scale bars = 200 µm.

We subsequently investigated acellular PLG scaffold transplantation into cynomolgus monkeys. Scaffolds were fabricated with a 35 mm diameter and 1:30 PLG:NaCl ratio, sterilized by γ irradiation, and implanted in the omentum (Figure 2A,B). Necropsy revealed no abnormal fibrotic tissue in the omentum, and the liver, gallbladder, and surrounding viscera appeared healthy in each animal (Figure 2C). Immunofluorescent staining of explanted PLG scaffolds and the omentum revealed the presence of fibroblasts, innate immune cells, and cytotoxic T cells in both tissues (Figure 2D and Supporting Information S2: Figure 2). H&E staining was performed on a section of the explanted scaffold, demonstrating host tissue integration with the acellular scaffold transplant (Figure 2D).

### 2.3 PLG Scaffolds Support Islet Survival in NHP

**Figure 3:**
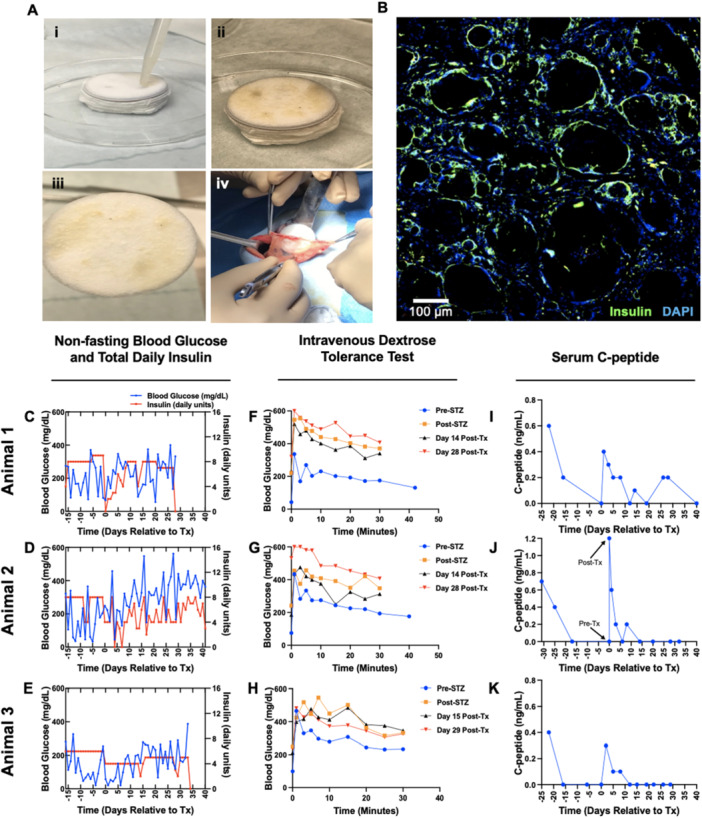
Allogeneic islet transplantation on irradiated scaffolds, fabricated with a 1:30 PLG:NaCl ratio, in the omentum of NHPs. (A) (i) Manual seeding of islets on the scaffold. (ii and iii) Scaffold after seeding with islets. (iv) Insertion of a cell‐laden scaffold in the omentum. (B) Immunohistochemical staining of an explanted scaffold from Animal 2, retrieved 41 days after transplantation. Insulin (green) and DAPI (blue) are colocalized throughout the image. Blank spaces are artifacts of processing and sectioning of such large tissues. Scale bar = 100 µm. (C–E) Timewise blood glucose and total daily exogenous insulin in diabetic NHP recipients. (F–H) IVDTTs were conducted before and after induction of diabetes with STZ, then again at serial timepoints after transplantation. (I–K) Serum C‐peptide levels were measured before and after induction of diabetes, then again after transplantation until termination of the study.

We next investigated the transplantation of allogeneic islets in STZ‐induced diabetic cynomolgus monkeys, with islets delivered on 35 mm irradiated scaffolds, fabricated with a 1:30 PLG:NaCl ratio (Figure 3A). The insulin regimen was adjusted between animals as indicated in Supporting Information S2: Tables 2 and 3. The initial recipient received 5326 IEQ/kg on one scaffold with exogenous insulin (Figure 3C,F,I). Exogenous insulin administration was reduced for the initial 12 days posttransplantation. Endogenous C‐peptide levels were initially 0.4 ng/mL and steadily declined from Day 1 to Day 12 (Figure 3C,I). C‐peptide was detected at levels of 0.2 ng/mL on Day 26 and Day 28 (the day of explant) before returning to 0 ng/mL on Day 40 (Figure 3I). Posttransplant intravenous dextrose tolerance tests (IVDTTs) showed no improvement in response compared to pretransplant in the first recipient (Figure 3F). During Day 14 and Day 28 IVDTTs, C‐peptide was detected at levels of 0.1 ng/mL, demonstrating impaired, but not absent, implant function (Supporting Information S2: Figure 1). The second recipient (Figure 3D,G,J) received 9409 IEQ/kg on one scaffold. C‐peptide was significantly elevated posttransplant yet was undetectable beyond Day 8 (Figure 3G,J). On Day 41, the animal was euthanized, and the scaffold was explanted for sectioning and staining (Figure 2D). Images showed evidence of islet survival, including colocalized insulin and DAPI (Figure 3B). The final recipient (Figure 3E,H,K) received 34,186 IEQ/kg on two scaffolds with a static insulin regimen (Supporting Information S2: Table 2). This animal exhibited the lowest mean glucose throughout the study (135.5 ± 22.059 mg/dL pretransplant, 171.4 ± 15.304 mg/dL posttransplant) and had reduced insulin requirements posttransplant relative to pretransplant. Similar to Animal 2, no detectable C‐peptide was identified after Day 8 in labs or IVDTTs. The animal was euthanized on Day 33, and the scaffold was explanted for sectioning and staining (Supporting Information S2: Figure 3).

An observation during this study was that the scaffold became fragile after seeding the islets, which had not been observed in prior studies with either mouse or NHP models (Gibly et al. 2011, 2013; Blomeier et al. 2006; Kasputis et al. 2018; Youngblood et al. 2019). Scaffolds maintained their structural integrity during and after γ irradiation, with no change in color or overall appearance upon visual inspection prior to seeding. This fragility made implantation challenging and may have impacted engraftment and function posttransplantation, which led to studies investigating scaffold fabrication.

### 2.4 Increasing the Polymer Content to Compensate for γ Irradiation

**Figure 4:**
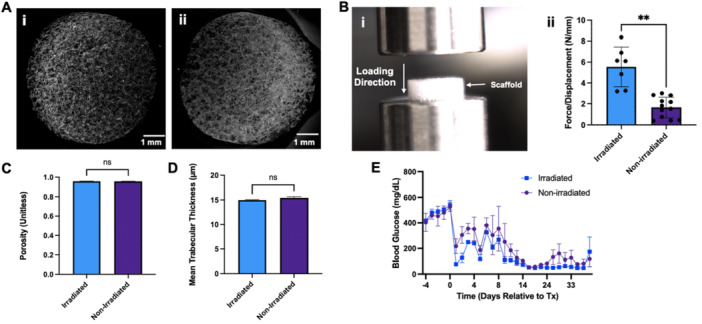
Characterization of γ‐irradiated scaffolds versus nonsterilized scaffolds, fabricated with a 1.25:30 PLG:NaCl ratio. (A) Representative µCT images of (i) a γ‐irradiated scaffold and (ii) a nonirradiated scaffold, visualized using the ImageJ2 3D Viewer. (B) Compressive testing of irradiated and nonirradiated PLG scaffolds. (i) Photograph of testing setup. (ii) The slope of the force–displacement curve was measured over a defined displacement interval (beginning at 0.1 mm) for each specimen, with statistical differences determined using a two‐tailed *t* test with Welch's correction (**p < 0.01). (C) Porosity of γ‐irradiated and nonirradiated scaffolds (*n* = 3 scaffolds per condition, each measured in triplicate). (D) Mean trabecular thickness (i.e., thickness of interior PLG branches) in γ‐irradiated and nonirradiated scaffolds (*n* = 3 scaffolds per condition, each measured in triplicate). (E) Blood glucose tracking for mice receiving 4000 IEQ (±20%) NHP islets on irradiated and nonirradiated scaffolds (*n* = 2 per condition).

The observed scaffold fragility was addressed by increasing the ratio of PLG:NaCl from 1:30 to 1.25:30 w/w. After irradiation, scaffolds remained uniform in appearance (Figure 4A), with pores of expected size visualized throughout the scaffold when observing cross‐sections. Irradiated scaffolds required significantly higher compressive force to deform than nonsterilized scaffolds, indicating an increase in stiffness (Figure 4B). As expected, scaffolds maintained a porosity above 95% (Figure 4C). The trabecular thickness, that is, the thickness of branches throughout the scaffold, was nearly identical before and after irradiation, averaging ~15 µm (Figure 4D) with low variance throughout the network.

We compared NHP islet transplant outcomes between mice receiving irradiated and nonirradiated scaffolds, fabricated with the increased ratio. Each mouse (*n* = 2 per condition) received 4000 IEQ (±20%). No significant difference in blood glucose levels over time was observed between conditions, and all mice were euglycemic by Day 12 posttransplantation (Figure 4E).

**Figure 5:**
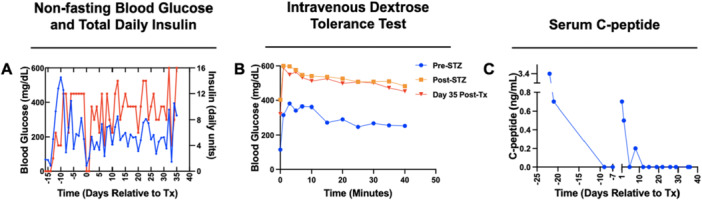
Pilot study of 1.25:30 PLG:NaCl scaffold for use in allogeneic islet transplantation in a NHP model. (A) Timewise blood glucose and total daily exogenous insulin. (B) IVDTTs. (C) Serum C‐peptide.

We next conducted a pilot study of the 1.25:30 PLG:NaCl scaffold in a diabetic NHP transplant recipient. Exogenous insulin administration was adjusted as indicated in Supporting Information S2: Tables 2 and 3, with this animal receiving the most aggressive regimen. This recipient received a dose of 22,083 IEQ/kg on one scaffold. Posttransplant, we observed high glucose variability similar to the initial diabetic recipients, and only slight decreases in insulin administration (Figure 5A). The posttransplant IVDTT had no indications of implant function, and C‐peptide was initially present posttransplantation yet not detected after Day 8 (animal was euthanized on Day 33).

### 2.5 PLG:NaCl Ratio Adjustment Compensates for Irradiation Without Compromising Islet Survival

**Figure 6:**
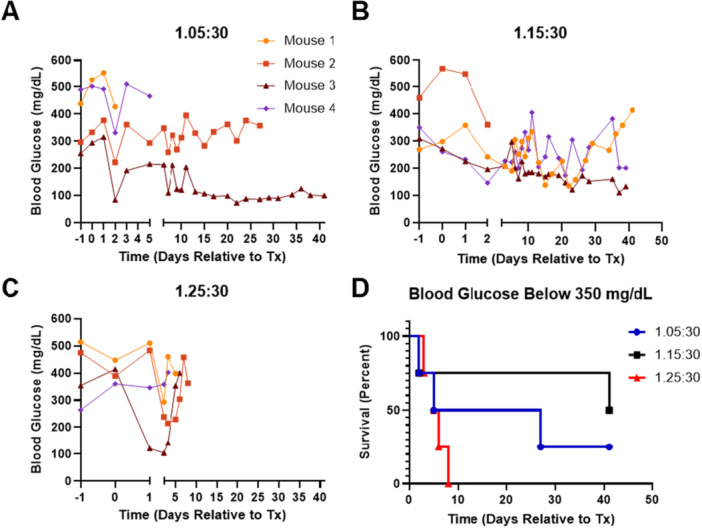
Further adjustment of PLG:NaCl ratio for xenogeneic islet transplants on irradiated scaffolds in NSG mice. Fasting blood glucose levels for individual mice, which received 2000 IEQ (±20%) of human islets on scaffolds fabricated with a (A) 1.05:30, (B) 1.15:30, or (C) 1.25:30 w/w PLG:NaCl ratio (*n* = 4 per condition). Once a mouse had two consecutive blood glucose readings ≥ 350 mg/dL, the mouse was eliminated from the study. (D) Reflecting this criterion, all mice are displayed in a Kaplan–Meier survival curve showing maintenance of fasting blood glucose below 350 mg/dL.

Lastly, we investigated two intermediate PLG:NaCl ratios in comparison to the 1.25:30 ratio in a mouse model. Four NSG mice per condition received a 2000 IEQ (±20%) total dose of human islets on irradiated scaffolds and were monitored for 40 days. If a mouse had two consecutive fasting blood glucose readings above 350 mg/dL, the transplant was considered to be unsuccessful, and the mouse was removed from the study. Mice receiving islets on 1.25:30 scaffolds exhibited the poorest maintenance of glucose below 350 mg/dL, with all four animals eliminated from the study by Day 8 (Figure 6C,D). Recipients of 1.05:30 scaffolds also exhibited relatively poor implant survival, though one mouse had complete restoration of euglycemia beginning ~1 week posttransplantation (Figure 6A,D). The 1.15:30 condition achieved the highest implant survival rate, with three out of four mice having maintained glucose below 350 mg/dL for more than a month after transplantation (Figure 6B,D). At least one mouse in this condition achieved euglycemia, with a second mouse exhibiting a trend toward long‐term glycemic control (Figure 6B).

## 3 Discussion

The widespread use of T1D cell therapies is hindered by several factors, including the hostility of the intrahepatic site, the requirement of systemic immunosuppression, and the scarcity of donor islets (Parums 2023; Qi et al. 2014; Czarnecka et al. 2023; Naziruddin et al. 2014; Brusko et al. 2021). We have previously shown that microporous PLG scaffolds seeded with islets can be implanted into the intraperitoneal fat, achieving euglycemia and insulin independence in murine models (Gibly et al. 2011; Blomeier et al. 2006; Gibly et al. 2013; Kasputis et al. 2018; Youngblood et al. 2019). In this work, we investigated the translatability of our approach in NHPs, using irradiated scaffolds with increased diameter to tighten sterility requirements and accommodate high cell volume. The acellular scaffolds implanted into the omentum of the NHP appeared well‐tolerated. Islets transplanted on these scaffolds in NHPs were identified by immunostaining, yet function was lower than anticipated relative to previous work with transplantation in mice.

Motivated by our previous work, we studied extrahepatic transplantation in NHPs as a test of scaffold performance in large animals with clinically relevant insulin requirements. A previous porcine study assessed islet survival, rather than therapeutic efficacy, on scaffolds in the omentum using densities that represent those needed for translation (Gibly et al. 2011). We observed effective islet revascularization in the porcine model when seeding at a density of ~15 IEQ/mm^3^ on 13 mm diameter scaffolds (~4000 IEQ/scaffold); in the NHP model, if we use Deng et al.'s average of 101,500 IEQ/transplant as a benchmark for full insulin independence, we would need three to four 35 mm diameter scaffolds to achieve that seeding density (Lei et al. 2022). NHP animals have been shown to have increased insulin requirements compared to humans (Casu et al. 2007). Additionally, NHP transplant recipients generally require a higher dose of islets to achieve insulin independence with immunosuppression relative to what is needed for humans (Czarnecka et al. 2023; Berman et al. 2016; Deng et al. 2023). Our studies identified the presence of surviving islets within the transplanted scaffolds, yet C‐peptide was detected in only one recipient near the end of the study period. C‐peptide levels declined below normal levels (0.51–2.72 ng/mL) typically within a week of transplantation. Previous studies with islet transplantation have reported similar declines in endogenous C‐peptide levels during the peritransplant period, with a late‐term increase beginning ~1‐month posttransplant at the earliest, which was the approximate duration of our study (Berman et al. 2009, 2016; Baidal et al. 2017; Deng et al. 2023).

Having previously shown islet survivability on larger‐scale scaffolds in a porcine model, we hypothesized that a further increase of scaffold size to adjust to NHP insulin requirements would achieve similar engraftment, independent of the glycemic outcomes. While nonirradiated 5 mm scaffolds used in mice maintained their integrity during seeding and transplantation, we observed greater fragility in 35 mm scaffolds, which we deduced was due to γ irradiation adversely impacting polymer stability (Krug et al. 2023; Montanari et al. 1998; Dorati et al. 2008; Makadia and Siegel 2011; Keles et al. 2015). Increasing the scaffold thickness was one option to increase stability, yet an increased thickness could also prolong cell infiltration and delay engraftment. Alternatively, modulating the PLG:NaCl ratio during fabrication can provide increased stability with γ irradiation, yet may impact the pore interconnectivity that influences host integration. In mouse models, we identified that a ratio of 1.15:30 maximally supported islet function in a cohort of mice receiving the same islet dose from the same donor. The 1.15:30 ratio demonstrated sustained glucose regulation in three of four mice (Figure 6D), without the postirradiation fragility present at lower ratios.

These studies were performed with the delivery of islets into the omentum and systemic immunosuppression, and this combination can be a factor influencing islet viability and function. Our NHP immunosuppression protocol bore several differences compared to other studies in the literature, including a distinct induction regimen (anti‐IgG1κ treatment and TNF inhibition) compared to Deng et al. (2023) (antithymocyte globulin and anti‐CD20) and Berman et al.'s (2016) study (antithymocyte globulin and belatacept). We also imposed a stricter limit on blood sirolimus concentrations (15 ng/mL) than Deng et al. (2023) (20 ng/mL). These agents pose an intrinsic risk to the islets due to drug toxicity, yet may not completely block immune cell compartments involved in fibrosis and adaptive immunity, which are present in the omentum (Naziruddin et al. 2014; Meza‐Perez and Randall 2017). Ultimately, our nonencapsulating approach may benefit from a localized immune modulation strategy, such as use of FasL‐ or PD‐L1‐functionalized biomaterials, that can protect allogeneic cells while allowing integration of the islets with surrounding tissue (Lei et al. 2022; Coronel et al. 2020; Li et al. 2023; Li et al. 2022; De Toni et al. 2022; Bochenek et al. 2018). Future studies could evaluate peritransplant management to establish unassisted glycemic control, as a nonenscapsulated approach can stress islets and diminish their function.

In conclusion, microporous PLG scaffolds have been successfully applied for islet transplantation in mouse and pig models, and herein we investigated the creation of large scaffolds for delivery in an NHP. Acellular PLG scaffolds were well tolerated in healthy NHPs, and islets transplanted on scaffolds were observed by histological staining. γ irradiation was employed for sterilization, which compromised the integrity of the large scaffolds and was associated with low levels of C‐peptide and a lack of glycemic control. A compensatory increase in the PLG:NaCl ratio may be able to balance the needs for structural stability while maintaining the survival and engraftment of the cells, though alternative sterilization methods should also be investigated. Additional studies could investigate alternative sterilization procedures, such as lower doses of γ irradiation or ethylene oxide, or additional modifications to scaffold composition and manufacturing. Scaffold modifications to enable long‐term gain of function in transplanted islets at extrahepatic sites have the potential to expand the population of eligible transplant recipients through alternative immune modulation strategies and cell sources (Lei et al. 2022; Coronel et al. 2020; Li et al. 2023; Li et al. 2022; Hogrebe et al. 2020; 2021).

## 4 Methods

### 4.1 Scaffold Fabrication and Sterilization

Scaffolds were fabricated using a salt porogen leaching method, which we have previously described (Youngblood et al. 2019; Nof and Shea 2002). The PLG:NaCl ratio was adjusted according to Supporting Information S2: Table 1, with scaffold diameters of 5 and 35 mm used for murine and NHP studies, respectively. Scaffolds used in the murine irradiated versus nonirradiated study were pressed as 35 mm scaffolds, with 5 mm sections taken with a 5 mm biopsy punch for subsequent murine transplants (denoted punched out in Supporting Information S2: Table 1). This strategy was used so murine transplants could be performed using scaffolds manufactured in the same manner as the NHP transplants. Scaffolds allocated for γ irradiation were sent to Steris Industries in sterilization pouches and received a dose of 43.80–45.09 kGy, for an exposure time of 522 min. After sterilization, sealed pouches were stored in a desiccator until the time of transplantation or material testing. Scaffolds used in NHP studies were subjected to endotoxin testing to demonstrate compliance with FDA guidelines for implanted medical devices (< 20 EU/device without blood contact).

### 4.2 Islet Isolation, Shipment, and Culture

All animal procedures pertinent to NHPs were performed in accordance with the Guidelines for Care and Use of Laboratory Animals of the University of Illinois‐Chicago (UIC, PRO21‐198 and PRO19‐013) and approved by the Animal Ethics Committee (IACUC) of UIC. NHP islets were isolated from the pancreata of deceased cynomolgus monkeys using collagenase (Sigma) and cultured overnight. Samples of the isolated islets were assessed with a viability stain and glucose‐stimulated insulin secretion assay to confirm function. For mouse studies, after isolation, islets were shipped overnight, where they were resuspended in CMRL 1066 medium (Corning) supplemented with 10% FBS and 1% ITS. Human islets were obtained from Prodo Laboratories Inc., where they underwent viability and purity testing prior to shipment. Upon receipt, they were cultured in PIM(S) media supplemented with PIM(ABS) and PIM(G) (Prodo Labs, Cat Nos. PIM‐S001GMP, PIM‐ABS001GMP, PIM‐G001GMP). All islets were cultured for a minimum of 3 h at 37°C and 5% CO_2_ to recover from transport.

### 4.3 NSG Mouse Studies

All murine studies were conducted in accordance with institutional guidelines and protocols (PRO00009714) approved by the University of Michigan IACUC. Naive NOD.Cg‐Prkdc^scid^Il2rg^tm1Wjl^/Szj (NSG) mice (Jackson Laboratories, Cat. No. 005557), 6–8 weeks of age, received intraperitoneal (IP) injections of STZ (Sigma, Cat. No. S0130) at a dose of 140 mg/kg to induce diabetes. Fasted blood glucose measurements were taken daily using an Accu‐Chek glucose meter (Roche Diabetes Care Inc., Indianapolis, IN). Mice with at least two consecutive fasting blood glucose readings above 350 mg/dL and less than a 20% loss in bodyweight compared to the day of injection were considered candidates for transplantation. Scaffolds were seeded with islets using large bore 200 µL pipette tips and then transplanted into the peritoneal fat as previously described (Bealer et al. 2023). Fasting blood glucose was measured daily for the first 10 days after transplantation, then three times per week for the duration of the studies. Nonsurvival explants were conducted by removing the fat pads after euthanasia, and survival explants were conducted following the same surgical procedure as transplantation.

### 4.4 Implantation of Acellular, Irradiated Scaffolds in NHPs to Assess Immunoreactivity

Three healthy cynomolgus monkeys (*Macaca fascicularis*) were sedated according to approved IACUC protocols. To prepare the surgical site, the fur was shaved from the xiphoid process to the pubis. The site (per‐umbilical and right and left upper quadrants) was then disinfected with betadine and isopropyl alcohol in triplicate.

Two irradiated, acellular, 35 mm scaffolds, fabricated with a 1:30 PLG:NaCl ratio, were gently inserted into a pocket of the bursa omentalis, opened with a single incision, using a spatula. Neither the scaffolds nor the incision were secured with sutures to avoid additional manipulation of the tissue that could cause fibrotic scarring. Animals were administered intravenous (IV) hydromorphone (0.1 mg/kg) and lidocaine (1.5–3.0 mg/kg/h) during surgery as needed. Bupivicaine (1 mg/kg, SQ) was administered at the end of the procedure before closure.

The bursa omentalis was removed from each recipient ~30 days after implantation and examined for signs of fibrosis and degradation of the scaffolds. Scaffold explants were isolated from the adipose tissue and either fixed in formalin for paraffin sectioning or flash frozen in isopentane and embedded in optimal cutting temperature compound for cryosectioning. Sectioned tissues were stained for α‐smooth muscle actin, a fibroblast marker; CD11b, a marker for neutrophils, monocytes, macrophages, and natural killer cells; and CD8, a marker for cytotoxic T cells. DAPI was used as a nuclear counterstain.

### 4.5 Allogeneic Islet Transplantation in Diabetic NHPs

The full NHP transplant procedure is described in Supporting Information S1: Supplemental Methods 1. In brief, diabetes was induced in four cynomolgus monkeys via 100 mg/kg IV STZ. Animals received exogenous insulin according to Supporting Information S2: Tables 2 and 3. Systemic immunosuppression began 1–2 weeks prior to surgery, and was monitored via biweekly blood draws.

NHP islets were isolated, cultured, aliquoted, and seeded onto 35 mm diameter, irradiated scaffolds following a similar procedure to as in the NSG mouse transplants. Dosing was adjusted for each animal, and scaffolds were delivered to the omentum of each recipient as described in Supporting Information S1: Supplemental Methods 1 and in accordance to IACUC protocols. Animals 1, 2, and 3 received islets on scaffolds fabricated with a 1:30 w/w PLG:NaCl ratio, and Animal 4 received islets on a scaffold fabricated with a 1.25:30 w/w PLG:NaCl ratio. Study endpoints ranged from 28 to 40 days after transplant.

### 4.6 Comparison of Nonsterilized and γ Irradiated Scaffolds With µCT

Irradiated and nonirradiated 5 mm diameter, 1.25:30 PLG:NaCl ratio scaffolds were imaged using a Nanotom M (Phoenix X‐ray, Waygate Technologies USA, Houston, TX). The X‐ray tube was fitted with a diamond‐coated tungsten target and 0.254 mm aluminum filter and powered to 65 kV and 250 µA. Spot size was set to zero. Imaging was done at 4 µm voxel size using an exposure time of 500 ms, with three frames averaged and one skipped for each rotation. The sample stage rotated through 360° and collected 2000 images/scan. Image acquisition and reconstruction of raw data were performed using Phoenix Datos|x 2 version 2.6.1 (Phoenix X‐ray, Waygate Technologies USA, Houston, TX).

Raw data files for each individual scaffold were imported into ImageJ2 (v. 2.14.0), and the BoneJ2 plugin was used for image analysis (Doube et al. 2010; Domander et al. 2021). Representative 3D µCT images were developed using the 3D Viewer plugin. Three scaffolds were imaged per condition, with porosity and mean branch thickness measurements done in triplicate, using three distinct regions.

### 4.7 Compressive Testing of Irradiated and Nonirradiated Scaffolds

Irradiated and nonirradiated scaffolds were subjected to compressive loading using a Mach‐1 Mechanical Testing System (Biomomentum Inc., Laval, QC, CA) on an anti‐vibration table. Scaffolds were transferred onto a steel compression platen of 9.53 mm diameter and centered to prevent torque during compression. An upper platen of the same diameter was lowered with a 0.005 mm/s ramp rate to establish contact with the sample (a load of 0.1015 N). Upon contact, the platen was raised to reach 2x the load resolution of the load cell, and relative displacement was set to zero. After 30 s for equilibration, the scaffold was compressed between the parallel platens at a 0.005 mm/s displacement rate until failure or a maximum displacement of 0.5 mm. Data acquisition was performed using the Mach‐1's mating software. For each scaffold, the slope of the force–displacement curve (*∆F/∆l*) was measured as an average across the linear region of the graph, typically beginning around a displacement of 0.1 mm.

### 4.8 Statistical Analysis

Statistical analyses were conducted using Prism 9.1.0 (GraphPad Software Inc.), with differences determined using a two‐tailed *t* test, unless otherwise noted. A significance level *α* = 0.05 was used for all studies. Significance is denoted as *p ≤ 0.05, **p ≤ 0.01, ***p ≤ 0.001, or ****p ≤ 0.0001. Values are reported as the mean ± standard error of the mean (SEM), unless otherwise noted. *n* denotes the number of biological replicates (animals) or the number of scaffolds analyzed (characterization studies).

## Author Contributions

Studies were conceived by Jessica L. King, Christopher Spencer, Richard Youngblood, Lonnie D. Shea, Peter D. Rios, and José Oberholzer. NSG mouse studies were performed by Jessica L. King, Richard Youngblood, Elizabeth Bealer, and Kelly Crumley. NHP studies were performed and overseen by Peter D. Rios, Ira Joshi, Sofia Ghani, Douglas Isa, James J. McGarrigle, David Cook, and José Oberholzer. Scaffold material characterization experiments were conceived and performed by Christopher Spencer, Conor Locke, Adam Abraham, and Andrea Clark. Jessica L. King, Christopher Spencer, and Lonnie D. Shea drafted and revised the manuscript.

## Conflicts of Interest

CellTrans is developing an islet product for transplantation as a therapy for type 1 diabetes.

## Supporting information

Supplemental Methods 1.

**Supplemental Figure 1:** Serum C‐peptide measurements taken during IVDTTs for Animal 1. **Supplemental Figure 2:** Technical replicates of sections from the PLG scaffold taken from the acellular scaffold transplant for α‐SMA (fibroblasts), CD11b (macrophages), and CD8 (cytotoxic T cells). Row two contains the full images containing the sections used in Figure 2D. **Supplemental Figure 3:** Representative staining of sections from the PLG scaffold taken from Animal 3 for α‐SMA (fibroblasts) and CD11b (macrophages). All scale bars = 500 µm. **Supplemental Table 1:** Summary of scaffold conditions included in this study. Variable parameters are listed in each column. The range for PLG weight includes the upper 5% error, since having too little PLG is detrimental to scaffold integrity; the range for NaCl weight includes the lower 5% error. **Supplemental Table 2:** Summary of insulin regimens used for each NHP transplant. *See Table 3. **Supplemental Table 3:** Sliding scales used for post‐transplant insulin administration for Animals 1, 2, and 4.

## Data Availability

The data that support the findings of this study are available from the corresponding author upon reasonable request.

## References

[bit70134-bib-0001] American Diabetes Association . 2023. *CGM and Time in Range*. American Diabetes Association. https://diabetes.org/tools-support/devices-technology/cgm-time-in-range#:~:text=Most%20people%20with%20type%201,Some%20may%20have%20different%20targets.

[bit70134-bib-0002] Arifin, D. R. , S. Valdeig , R. A. Anders , J. W. M. Bulte , and C. R. Weiss . 2016. “Magnetoencapsulated Human Islets Xenotransplanted Into Swine: A Comparison of Different Transplantation Sites.” Xenotransplantation 23: 211–221. 27225644 10.1111/xen.12235 PMC5027196

[bit70134-bib-0003] Baidal, D. A. , C. Ricordi , D. M. Berman , et al. 2017. “Bioengineering of an Intraabdominal Endocrine Pancreas.” New England Journal of Medicine 376: 1887–1889. 28489987 10.1056/NEJMc1613959 PMC5572072

[bit70134-bib-0004] Bealer, E. , K. Crumley , D. Clough , et al. 2023. “Extrahepatic Transplantation of 3D Cultured Stem Cell‐Derived Islet Organoids on Microporous Scaffolds.” Biomaterials Science 11: 3645–3655. 37017294 10.1039/d3bm00217a PMC10192035

[bit70134-bib-0005] Berman, D. M. , R. D. Molano , C. Fotino , et al. 2016. “Bioengineering the Endocrine Pancreas: Intraomental Islet Transplantation Within a Biologic Resorbable Scaffold.” Diabetes 65: 1350–1361. 26916086 10.2337/db15-1525 PMC5384628

[bit70134-bib-0006] Berman, D. M. , J. J. O'Neil , L. Coffey , et al. 2009. “Long‐Term Survival of Nonhuman Primate Islets Implanted in an Omental Pouch on a Biodegradable Scaffold.” American Journal of Transplantation 9: 91–104. 19133931 10.1111/j.1600-6143.2008.02489.x PMC4441095

[bit70134-bib-0007] Blomeier, H. , X. Zhang , C. Rives , et al. 2006. “Polymer Scaffolds as Synthetic Microenvironments for Extrahepatic Islet Transplantation.” Transplantation 82: 452–459. 10.1097/01.tp.0000231708.19937.21. 16926587 PMC2648394

[bit70134-bib-0008] Bochenek, M. A. , O. Veiseh , A. J. Vegas , et al. 2018. “Alginate Encapsulation as Long‐Term Immune Protection of Allogeneic Pancreatic Islet Cells Transplanted Into the Omental Bursa of Macaques.” Nature Biomedical Engineering 2: 810–821. 10.1038/s41551-018-0275-1 PMC6413527 30873298

[bit70134-bib-0009] Brusko, T. M. , H. A. Russ , and C. L. Stabler . 2021. “Strategies for Durable β Cell Replacement in Type 1 Diabetes.” Science 373: 516–522. 34326233 10.1126/science.abh1657 PMC8867839

[bit70134-bib-0010] Casu, A. , R. Bottino , A. N. Balamurugan , et al. 2007. “Metabolic Aspects of Pig‐to‐Monkey (*Macaca fascicularis*) Islet Transplantation: Implications for Translation Into Clinical Practice.” Diabetologia 51: 120–129. 17960359 10.1007/s00125-007-0844-4

[bit70134-bib-0011] ClinicalTrials.gov . 2023a. “A Safety, Tolerability, and Efficacy Study of VX‐880 in Participants With Type 1 Diabetes.” https://classic.clinicaltrials.gov/ct2/show/NCT04786262?term=VX-880&draw=2&rank=1.

[bit70134-bib-0012] ClinicalTrials.gov . 2023b. “A Safety, Tolerability, and Efficacy Study of Sernova's Cell Pouch for Clinical Islet Transplantation.” https://clinicaltrials.gov/study/NCT03513939?intr=Sernova%20Cell%20Pouch&rank=1.

[bit70134-bib-0013] Coronel, M. M. , K. E. Martin , M. D. Hunckler , et al. 2020. “Immunotherapy via PD‐L1‐Presenting Biomaterials Leads to Long‐Term Islet Graft Survival.” Science Advances 6: eaba5573. 32923626 10.1126/sciadv.aba5573 PMC7455180

[bit70134-bib-0014] Czarnecka, Z. , N. Dadheech , H. Razavy , R. Pawlick , and A. M. J. Shapiro . 2023. “The Current Status of Allogenic Islet Cell Transplantation.” Cells 12: 2423. 37887267 10.3390/cells12202423 PMC10605704

[bit70134-bib-0015] De Toni, T. , A. A. Stock , F. Devaux , et al. 2022. “Parallel Evaluation of Polyethylene Glycol Conformal Coating and Alginate Microencapsulation as Immunoisolation Strategies for Pancreatic Islet Transplantation.” Frontiers in Bioengineering and Biotechnology 10: 886483. 35651551 10.3389/fbioe.2022.886483 PMC9149081

[bit70134-bib-0016] Deng, H. , A. Zhang , D. Pang , et al. 2023. “Bioengineered Omental Transplant Site Promotes Pancreatic Islet Allografts Survival in Non‐Human Primates.” Cell Reports Medicine 4: 100959. 36863336 10.1016/j.xcrm.2023.100959 PMC10040375

[bit70134-bib-0017] Domander, R. , A. A. Felder , and M. Doube . 2021. “BoneJ2 ‐ Refactoring Established Research Software.” Wellcome Open Research 6: 37. 33954267 10.12688/wellcomeopenres.16619.1 PMC8063517

[bit70134-bib-0018] Dorati, R. , C. Colonna , M. Serra , et al. 2008. “γ‐Irradiation of PEGd, lPLA and PEG‐PLGA Multiblock Copolymers: I. Effect of Irradiation Doses.” AAPS PharmSciTech 9: 718. 18528761 10.1208/s12249-008-9103-3 PMC2976929

[bit70134-bib-0019] Doube, M. , M. M. Kłosowski , I. Arganda‐Carreras , et al. 2010. “BoneJ: Free and Extensible Bone Image Analysis in ImageJ.” Bone 47: 1076–1079. 20817052 10.1016/j.bone.2010.08.023 PMC3193171

[bit70134-bib-0020] Gibly, R. F. , X. Zhang , M. L. Graham , et al. 2011. “Extrahepatic Islet Transplantation With Microporous Polymer Scaffolds in Syngeneic Mouse and Allogeneic Porcine Models.” Biomaterials 32: 9677–9684. 21959005 10.1016/j.biomaterials.2011.08.084 PMC3195897

[bit70134-bib-0021] Gibly, R. F. , X. Zhang , W. L. Lowe , and L. D. Shea . 2013. “Porous Scaffolds Support Extrahepatic Human Islet Transplantation, Engraftment, and Function in Mice.” Cell Transplantation 22: 811–819. 22507300 10.3727/096368912X636966 PMC3701739

[bit70134-bib-0022] Gregory, G. A. , T. Robinson , S. E. Linklater , et al. 2022. “Global Incidence, Prevalence, and Mortality of Type 1 Diabetes in 2021 With Projection to 2040: A Modelling Study.” Lancet Diabetes & Endocrinology 10: 741–760. 36113507 10.1016/S2213-8587(22)00218-2

[bit70134-bib-0024] Hogrebe, N. J. , P. Augsornworawat , K. G. Maxwell , L. Velazco‐Cruz , and J. R. Millman . 2020. “Targeting the Cytoskeleton to Direct Pancreatic Differentiation of Human Pluripotent Stem Cells.” Nature Biotechnology 38: 460–470. 10.1038/s41587-020-0430-6 PMC7274216 32094658

[bit70134-bib-0025] Hogrebe, N. J. , K. G. Maxwell , P. Augsornworawat , and J. R. Millman . 2021. “Generation of Insulin‐Producing Pancreatic β Cells From Multiple Human Stem Cell Lines.” Nature Protocols 16: 4109–4143. 34349281 10.1038/s41596-021-00560-y PMC8529911

[bit70134-bib-0026] Hunckler, M. D. , and A. J. García . 2020. “Engineered Biomaterials for Enhanced Function of Insulin‐Secreting β‐Cell Organoids.” Advanced Functional Materials 30: 2000134.

[bit70134-bib-0027] Joish, V. N. , F. L. Zhou , R. Preblick , et al. 2020. “Estimation of Annual Health Care Costs for Adults With Type 1 Diabetes in the United States.” Journal of Managed Care & Specialty Pharmacy 26: 311–318. 32105172 10.18553/jmcp.2020.26.3.311 PMC10390990

[bit70134-bib-0028] Kasputis, T. , D. Clough , F. Noto , K. Rychel , B. Dye , and L. D. Shea . 2018. “Microporous Polymer Scaffolds for the Transplantation of Embryonic Stem Cell Derived Pancreatic Progenitors to a Clinically Translatable Site for the Treatment of Type I Diabetes.” ACS Biomaterials Science & Engineering 4: 1770–1778. 10.1021/acsbiomaterials.7b00912. 30345348 PMC6191190

[bit70134-bib-0029] Keles, H. , A. Naylor , F. Clegg , and C. Sammon . 2015. “Investigation of Factors Influencing the Hydrolytic Degradation of Single PLGA Microparticles.” Polymer Degradation and Stability 119: 228–241.

[bit70134-bib-0030] Krug, N. , J.‐C. Zarges , and H.‐P. Heim . 2023. “Influence of Ethylene Oxide and Gamma Irradiation Sterilization Processes on the Properties of Poly‐l‐Lactic‐Acid (PLLA) Materials.” Polymers 15: 3461. 37631518 10.3390/polym15163461 PMC10458838

[bit70134-bib-0031] Lei, J. , M. M. Coronel , E. S. Yolcu , et al. 2022. “FasL Microgels Induce Immune Acceptance of Islet Allografts in Nonhuman Primates.” Science Advances 8: eabm9881. 35559682 10.1126/sciadv.abm9881 PMC9106299

[bit70134-bib-0032] Li, F. , K. Crumley , E. Bealer , et al. 2023. “FAS Ligand‐Modified Scaffolds Protect Stem Cell Derived β‐Cells by Modulating Immune Cell Numbers and Polarization.” ACS Applied Materials & Interfaces 15, 50549–50559. 36533683 10.1021/acsami.2c12939

[bit70134-bib-0033] Li, F. , F. Li , E. Bealer , et al. 2022. “Membrane‐Coated Nanoparticles for Direct Recognition by T Cells.” Biotechnology and Bioengineering 120, 767–777. 36515455 10.1002/bit.28304

[bit70134-bib-0034] Makadia, H. K. , and S. J. Siegel . 2011. “Poly Lactic‐co‐Glycolic Acid (PLGA) as Biodegradable Controlled Drug Delivery Carrier.” Polymers 3: 1377–1397. 22577513 10.3390/polym3031377 PMC3347861

[bit70134-bib-0035] Meza‐Perez, S. , and T. D. Randall . 2017. “Immunological Functions of the Omentum.” Trends in Immunology 38: 526–536. 28579319 10.1016/j.it.2017.03.002 PMC5812451

[bit70134-bib-0036] Montanari, L. , M. Costantini , E. C. Signoretti , et al. 1998. “Gamma Irradiation Effects on Poly(dl‐lactictide‐co‐glycolide) Microspheres.” Journal of Controlled Release 56: 219–229. 9801445 10.1016/s0168-3659(98)00082-0

[bit70134-bib-0037] Naziruddin, B. , S. Iwahashi , M. A. Kanak , M. Takita , T. Itoh , and M. F. Levy . 2014. “Evidence for Instant Blood‐Mediated Inflammatory Reaction in Clinical Autologous Islet Transplantation.” American Journal of Transplantation 14: 428–437. 24447621 10.1111/ajt.12558

[bit70134-bib-0038] Nof, M. , and L. D. Shea . 2002. “Drug‐Releasing Scaffolds Fabricated From Drug‐Loaded Microspheres.” Journal of Biomedical Materials Research 59: 349–356. 11745572 10.1002/jbm.1251

[bit70134-bib-0039] Parums, D. V. 2023. “Editorial: First Regulatory Approval for Allogeneic Pancreatic Islet Beta Cell Infusion for Adult Patients With Type 1 Diabetes Mellitus.” Medical Science Monitor 29: e941918. 37525584 10.12659/MSM.941918 PMC10403990

[bit70134-bib-0040] Qi, M. , K. Kinzer , K. K. Danielson , et al. 2014. “Five‐Year Follow‐Up of Patients With Type 1 Diabetes Transplanted With Allogeneic Islets: The UIC Experience.” Acta Diabetologica 51: 833–843. 25034311 10.1007/s00592-014-0627-6 PMC4801517

[bit70134-bib-0041] Stice, M. J. , T. B. Dunn , M. D. Bellin , M. E. Skube , and G. J. Beilman . 2018. “Omental Pouch Technique for Combined Site Islet Autotransplantation Following Total Pancreatectomy.” Cell Transplantation 27: 1561–1568. 30215272 10.1177/0963689718798627 PMC6180729

[bit70134-bib-0042] U.S. Food and Drug Administration . 2012. *Guidance for Industry: Pyrogen and Endotoxins Testing: Questions and Answers*. U.S. Food and Drug Administration. https://www.fda.gov/regulatory-information/search-fda-guidance-documents/guidance-industry-pyrogen-and-endotoxins-testing-questions-and-answers.

[bit70134-bib-0043] Williams, J. , N. Jacus , K. Kavalackal , et al. 2018. “Over Ten‐Year Insulin Independence Following Single Allogeneic Islet Transplant Without T‐Cell Depleting Antibody Induction.” Islets 10: 168–174. 30024826 10.1080/19382014.2018.1451281 PMC6281363

[bit70134-bib-0044] Youngblood, R. L. , J. P. Sampson , K. R. Lebioda , and L. D. Shea . 2019. “Microporous Scaffolds Support Assembly and Differentiation of Pancreatic Progenitors Into β‐cell Clusters.” Acta Biomaterialia 96: 111–122. 31247380 10.1016/j.actbio.2019.06.032 PMC6717676

[bit70134-bib-0050] Zajec, A. , K. Trebušak Podkrajšek , T. Tesovnik , et al. 2022. “Pathogenesis of Type 1 Diabetes: Established Facts and New Insights.” Genes 13: 706. 35456512 10.3390/genes13040706 PMC9032728

