# Supplementary material for: Gamma Irradiation of Poly(lactide‐co‐glycolide) Scaffolds Reduces the Mechanical Stability and Function of Islet Grafts in Diabetic Nonhuman Primates
Source: Biotechnol Bioeng. 2025 Dec 24;123(3):742–51. doi: 10.1002/bit.70134 (PMC12883903; doi:10.1002/bit.70134)
Supplement: Supplementary file 1 — Supplemental Methods 1. [file BIT-123-742-s001.docx]

Supplemental methods 1

In four separate pilot studies, a diabetic cynomolgus monkey received an allogeneic islet transplant via irradiated scaffolds implanted in the omentum. Each recipient was administered 100 mg/kg STZ (Teva Parenteral Medicines, Inc., Tel Aviv, Israel) via IV 15-24 days prior to surgery to induce diabetes. Animals received 150-200 mL of IV saline pre-STZ and 250 mL of IV saline post-STZ. Once diabetes was established, animals received long-acting exogenous insulin via SQ injection (Lantus 100 units/mL, Sanofi, Paris, France) throughout the duration of the study. The insulin regimen was adjusted for each animal (Supplemental Tables 2-3).

Candidacy for surgery was determined by measuring bodyweight and serum C-peptide before STZ (days -22, -21, and -16) and after STZ (days -14, -9, -7, -5, and -1). In addition, fasted intravenous dextrose tolerance tests (IVDTTs) were conducted before and after STZ, during which blood glucose and C-peptide were measured at serial timepoints up to 40 minutes after the dextrose challenge. Blood glucose was monitored using either an Accu-Chek glucose meter or the FreeStyle Libre 2 Continuous Glucose Monitor (Abbott Diabetes Care, Alameda, CA), with nonfasting readings taken every morning and evening.

Allogeneic islet transplant recipients were systemically immunosuppressed with tacrolimus (Prograf, Astellas Pharma, Inc., Northbrook, IL) and sirolimus (Rapamune, LC Laboratories, Woburn, MA). Immunosuppression was initiated approximately 1-2 weeks prior to transplantation with starting doses of 0.025 mg/kg tacrolimus and 0.020 mg/kg sirolimus, twice per day. Blood draws were performed two times per week to verify therapeutic concentrations of tacrolimus (4-6 ng/mL, measured by homogeneous particle-enhanced turbidimetric immunoassay) and sirolimus (10-15 ng/mL, measured by chemiluminescent microparticle immunoassay). Basiliximab (Simulect, Novartis, East Hanover, NJ) was administered as follows: 5 mg IV on Day 0, 25 mg IV on Day 14 and Day 28 post-transplant. Etanercept (Enbrel, Amgen, Thousand Oaks, CA) was administered as follows: 50 mg IV on Day 0, then 25 mg SQ on Day 3, Day 7±1, and Day 10±1.

NHP islets were isolated, cultured, aliquoted, and seeded onto 35 mm diameter, irradiated scaffolds following a similar procedure to as in the NSG mouse transplants. For Animal 1, a scaffold was seeded with 33,024 IEQ (5,326 IEQ/kg dose, taken from four donors) using a custom, vacuum-driven seeding device. For Animal 2 (9,409 IEQ/kg, six donors), Animal 3 (34,186 IEQ/kg, eight donors), and Animal 4 (22,083 IEQ/kg, four donors), islets were seeded manually using a large bore pipette tip. Animals 1, 2, and 3 received islets on scaffolds fabricated with a 1:30 w/w PLG:NaCl ratio, and Animal 4 received islets on a scaffold fabricated with a 1.25:30 w/w PLG:NaCl ratio.

On the day of surgery, each transplant recipient was sedated and anesthetized per IACUC protocols and in the same manner as the acellular scaffold transplant, and Meloxicam SR (0.6 mg/kg, SQ) was administered prior to surgery. Under aseptic conditions, a midline incision (6-8 cm) was created using a sterile scalpel blade, before assessment of the intraperitoneal cavity. A 6 cm incision was made in the omentum. The omentum was then held with sterile surgical graspers or forceps while inserting the scaffold in between the layers of the omentum. No additional manipulation was needed to close the omentum incision. The abdominal incisions were closed in 3 layers using 3-0 Vicryl for the abdominal wall and subcutaneous layer, and 4-0 Vicryl for the skin (subcuticular). Buprenorphine (0.2 mg/kg, SQ) was administered immediately post-surgery. After transplant, blood glucose was monitored twice daily, in the same manner as pre-transplant. Long-acting exogenous insulin therapy was continued as shown in Supplementary Tables 2-3. Bodyweight and serum C-peptide were measured on the day of transplant (day 0) and twice per week thereafter.

Study endpoints ranged from 28-40 days after transplant. Each monkey was sedated and anesthetized as previously described, administered 500 U/kg heparin via IV injection 10 minutes prior to euthanasia, and euthanized with an overdose of sodium pentobarbital (1 mL/10 lb). A necropsy was performed, and the entire omentum was removed, with scaffolds retrieved from Animal 2 for cryosectioning. Tissue sections were stained for insulin and DAPI.
