# Supplementary material for: Gamma Irradiation of Poly(lactide‐co‐glycolide) Scaffolds Reduces the Mechanical Stability and Function of Islet Grafts in Diabetic Nonhuman Primates
Source: Biotechnol Bioeng. 2025 Dec 24;123(3):742–51. doi: 10.1002/bit.70134 (PMC12883903; doi:10.1002/bit.70134)
Supplement: Supplementary file 2 — Supplemental Figure 1: Serum C‐peptide measurements taken during IVDTTs for Animal 1. Supplemental Figure 2: Technical replicates of sections from the PLG scaffold taken from the acellular scaffold transplant for α‐SMA (fibroblasts), CD11b (macrophages), and CD8 (cytotoxic T cells). Row two contains the full images containing the sections used in Figure 2D. Supplemental Figure 3: Representative staining of sections from the PLG scaffold taken from Animal 3 for α‐SMA (fibroblasts) and CD11b (macrophages). All scale bars = 500 µm. Supplemental Table 1: Summary of scaffold conditions included in this study. Variable parameters are listed in each column. The range for PLG weight includes the upper 5% error, since having too little PLG is detrimental to scaffold integrity; the range for NaCl weight includes the lower 5% error. Supplemental Table 2: Summary of insulin regimens used for each NHP transplant. *See Table 3. Supplemental Table 3: Sliding scales used for post‐transplant insulin administration for Animals 1, 2, and 4. [file BIT-123-742-s002.docx]

**Supplemental information**

**
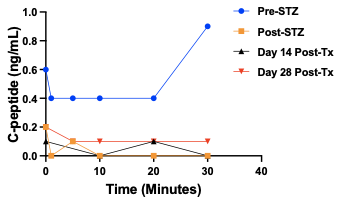
**

Supplemental Figure 1: Serum C-peptide measurements taken during IVDTTs for Animal 1.


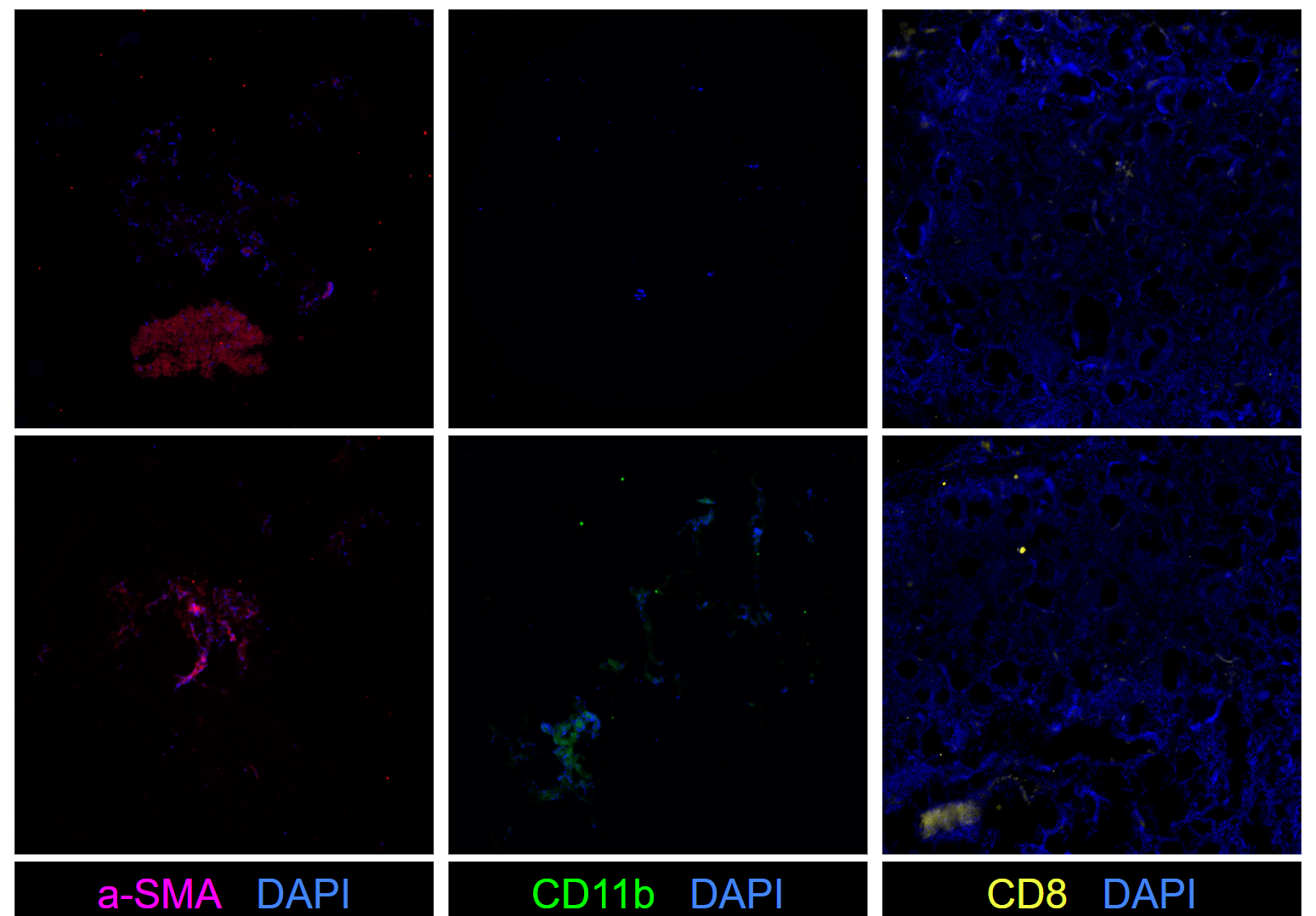
Supplemental Figure 2: Technical replicates of sections from the PLG scaffold taken from the acellular scaffold transplant for α-SMA (fibroblasts), CD11b (macrophages), and CD8 (cytotoxic T cells). Row two contains the full images containing the sections used in Figure 2D.


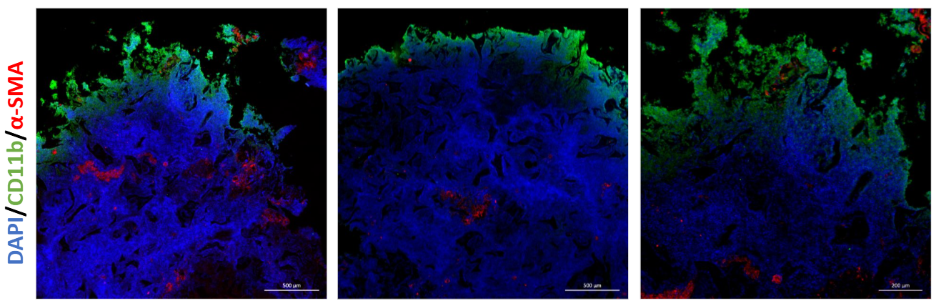


Supplemental Figure 3: Representative staining of sections from the PLG scaffold taken from Animal 3 for α-SMA (fibroblasts) and CD11b (macrophages). All scale bars = 500 µm.

Supplemental Table 1: Summary of scaffold conditions included in this study. Variable parameters are listed in each column. The range for PLG weight includes the upper 5% error, since having too little PLG is detrimental to scaffold integrity; the range for NaCl weight includes the lower 5% error.

| **Irradiated? (Y/N)** | **Diameter (mm)** | **PLG:NaCl Ratio (w/w)** | **PLG Weight (mg)** | **NaCl Weight (mg)** | **Experiments Conducted** |
| --- | --- | --- | --- | --- | --- |
| N | 5 | 1:30 | 2.5-2.625 | 71.25-75 | NHP/Human Islet Comparison |
| Y | 35 | 1:30 | 135-141.75 | 3,848-  4,050 | NHP Immunoreactivity, NHP Transplants |
| Y | 35 (punched out) | 1.25:30 | 168.75-  177.188 | 3,848-  4,050 | Murine Irradiated vs. Non-irradiated |
| N | 35 (punched out) | 1.25:30 | 168.75-  177.188 | 3,848-  4,050 | Murine Irradiated vs. Non-irradiated |
| Y | 35 | 1.25:30 | 153.125-  160.781 | 3,492-  3,675 | NHP Transplants |
| Y | 5 | 1.05:30 | 2.625-2.756 | 71.25-75 | PLG:NaCl Ratio Comparison |
| Y | 5 | 1.15:30 | 2.875-3.019 | 71.25-75 | PLG:NaCl Ratio Comparison |
| Y | 5 | 1.25:30 | 3.125-3.281 | 71.25-75 | PLG:NaCl Ratio Comparison, µCT, Compression |
| N | 5 | 1.25:30 | 3.125-3.281 | 71.25-75 | µCT, Compression |

Supplemental Table 2: Summary of insulin regimens used for each NHP transplant. *See Table 3.

| **Animal #** | **Days Administered**  **(Relative to Tx)** | **Morning Dose**  **(Units)** | **Evening Dose**  **(Units)** |
| --- | --- | --- | --- |
| 1 | -16 to -6 | 4 | 4 |
|  | -5 to -1 | 5 | 4 |
|  | 0 to 8 (morning) | Sliding Scale* | Sliding Scale* |
|  | 8 (evening) to 21 | 4 | 4 |
|  | 21 to 27 | 4 | 3 |
| 2 | -21 to -1 | 4 | 4 |
|  | 0 to 41 | Sliding Scale* | Sliding Scale* |
| 3 | -19 to -1 | 3 | 3 |
|  | 0 to 15 | 2 | 2 |
|  | 16 to 33 | 3 | 2 |
| 4 | -13 to 0 | 4-6 | 0-6 |
|  | 0 to 35 | Sliding Scale* | Sliding Scale* |

Supplemental Table 3: Sliding scales used for post-transplant insulin administration for Animals 1, 2, and 4.

| Animal 1 | | Animal 2 | | Animal 4 | |
| --- | --- | --- | --- | --- | --- |
| BG (mg/dL) | Insulin (Units) | BG (mg/dL) | Insulin (Units) | BG (mg/dL) | Insulin (Units) |
| ≤199 | 0 | ≤199 | 0 | ≤49 | 0 |
| 200-299 | 2 | 200-299 | 2 | 50-199 | 4 |
| 300-399 | 4 | 300-399 | 3 | 200-299 | 6 |
| 400-499 | 6 | ≥400 | 4 | ≥300 | 8 |
| ≥500 | 8 | – | – | – | – |
